# Editorial: Current advances in pediatric surgery

**DOI:** 10.3389/fsurg.2023.1161556

**Published:** 2023-03-21

**Authors:** Gunadi

**Affiliations:** Pediatric Surgery Division, Department of Surgery, Faculty of Medicine, Public Health and Nursing, Universitas Gadjah Mada, Yogyakarta, Indonesia

**Keywords:** cell-Based therapy and tissue engineering, congenital anomaly, advances on fetal surgery, genetic disorders, molecular genetics, minimally invasive & robotic surgery, organ transplantation, prenatal diagnosis

**Editorial on the Research Topic** Current advances in pediatric surgery

The rapid development of recent technologies and research in the world has helped advance medical care in terms of how we diagnose, give therapies, and predict disease prognosis. It also improves our understanding of diseases leading to more personalized medicine. These developments have undeniably improved surgical practice, especially in many pediatric surgery fields. However, there still needs to be more knowledge among physicians and academics worldwide about these advancements, especially in developing countries. This research topic provides space for clinicians, academics, or anyone who wishes to keep up to date with some advances in pediatric surgery. This research topic covered many pediatric surgery fields, including imaging, organ transplantation, minimally invasive surgery, robotic surgery, prognostic predictors, and others. In this editorial, we presented the summary of the papers based on the topics of diagnostic biomarkers, imaging techniques, non-surgical and surgical treatments, prognosis, and others.

The use of biomarkers are on the rise due to their advantages in early diagnosis and predicting outcomes. Therefore, many studies are evaluating the use of some biomarkers in diseases including pediatric surgery cases. A study in China analyzed the plasma amyloid-beta levels (Aβ42/Aβ40) as a biomarker for biliary atresia (BA)) and its correlation with hepatic dysfunctions. They found that Aβ42/Aβ40 is a good indicator for cholestasis but not enough for differentiating BA and non-BA. It must be combined with γ-glutamyl transpeptidase (GGT) and one other hepatic function parameter. This will enable early intraoperative cholangiography for BA confirmation and early treatment for better outcomes Lyu et al.

Proper imaging of the lesions in pediatric cases is essential to assess and deciding the most appropriate treatment. A report from the UK studied the use of micro-CT imaging in a pediatric thyroglossal duct Frauenfelder et al. They found that this imaging was helpful as a visual aid to traditional histopathological examination due to its 3D imaging capability. Another study evaluated the accuracy of hepatic scintigraphy in diagnosing biliary atresia as a cause of cholestatic jaundice. They concluded that this method is still limited in accuracy, and surgical exploration remains the gold standard Chan et al. Moreover, a study reported using indocyanine green (ICG) angiography to assess skin-sparing in acute pediatric trauma. This technique has been proven helpful in assessing skin flap perfusion in reconstructive surgeries. They found that this technique may decrease the risk of postoperative necrosis and can be a feasible adjuvant technique in skin-sparing decision- making in acute pediatric trauma Han et al.

In recent years, non-surgical and surgical techniques in pediatric cases have developed rapidly worldwide. Pediatric surgeons must keep up to date with the latest and most effective techniques which produce the best outcome with the current evidence. A retrospective study in the Netherlands analyzed the use of botulinum toxin injections in children with chronic constipation. They found that botulinum toxin significantly reduced anal basal pressure when preinjection pressure exceeds 70 mmHg. However, in severely elevated anal basal pressure, rectal washout is recommended Sun et al. Total splenectomy is the most effective treatment in a patient with moderate or severe hereditary spherocytosis (HS). Partial splenic embolization (PSE) is a conservative treatment that is able to preserve part of the spleen's function. A retrospective study in a single center reviewed the cases of HS treated with super- selective PSE (SPSE) and total splenectomy. They found that SPSE is safe and effective in moderate or severe pediatric HS; however, more patients are needed, along with longer follow- ups Wang et al.

Regarding surgical techniques in pediatric surgery patients, Ritz et al. reported five patients with esophageal perforation (EP) treated with endoscopic esophageal vacuum- assisted closure (EVAC), ArgyleTM Replogle Suction Catheter (RSC), or both. They found EVAC, widely used to treat wounds and adult patients with EP, to be a promising therapy for pediatric EP. They also recommended an earlier switch to RSC to reduce the use of anesthesia in subsequent treatments. After one year, most patients gained sufficient weight. Another case series in China Huang et al. found that debridement of pleural empyema using uniportal video-assisted thoracic surgery (U-VATS) is a feasible and effective surgical technique in pediatric stage II and III empyema. This technique provides easier use and complete debridement with a low risk for conversion. Moreover, a retrospective study in 86 children with severe proximal hypospadias with incomplete penoscrotal transposition found that a novel repair method using tunica vaginalis flap-covering combined with modified Glenn–Anderson in one-stage repair was a safe, effective, and simpler method Wang et al. Another study of limb osteosarcoma in pediatric patients found that amputation is associated with a 1.5-fold increase in cancer-specific mortality (CSM). Therefore, limb salvage surgery should be preferred as a first choice without other contraindications Wang et al.

The use of minimally invasive surgery in pediatric cases, if possible, has been preferred, with less operating and recovery time as the main advantages. Huang et al. reported using U-VATS in patients with pulmonary sequestration and found satisfactory perioperative results. A study in China reported on two infants with congenital hyperinsulinism of infancy (CHI) who underwent laparoscopic pancreatic head resection and Roux-en-Y pancreaticojejunostomy. They found this technique to be safe and effective with a good prognosis Wen et al. A study in the same institution introduced a novel technique for pediatric inguinal hernia (PIH) repair, called transumbilical single-site laparoscopic intraperitoneal closure (TUSLIC) of the internal inguinal ring (IIR) using a single instrument. This technique aimed to improve on the previous technique, such as transabdominal multiple-site laparoscopic extraperitoneal closure (TAMLEC) of IIR, which is known to cause some complications. They concluded that TUSLIC is a safe and reliable method for PIH, with lower complications and recurrence rates than TAMLEC Ji et al. With the rise of robotic surgery, its usage in pediatric surgery cases can also provide an alternative to otherwise complicated procedures with a high learning curve. A study in China reported using the da Vinci surgical system for choledochal cyst excision in children below one year old. They found that this system was safe and feasible to use Xie et al.

The need for new techniques in organ transplantation surgeries in pediatrics is also essential, and the reports of these, especially in pediatric cases, are still scarce. Wang et al. analyzed and compared the super minimal incision technique in pediatric kidney transplantation (SMIPKT) and conventional kidney transplantation (CKT). They found that SMIPKT produced better cosmetics, shorter operation time, and reduced bleeding and postoperative drainage volume within 24 h. Moreover, in terms of postoperative complications, no differences were found. This result could be an essential step forward in kidney transplantation in pediatric patients, and further study with a larger sample size is still needed. In another organ, there was a literature review and case report on using meso-Rex bypass (MRB) post-liver transplantation (LT). They concluded that MRB post-LT is a challenging procedure and needs careful planning. However, in a patient with advanced complications, this can benefit from autologous IJV graft and have adequate recovery Dalzell et al.

After any treatment, it is imperative to predict the outcomes or prognosis of the patients. Early detection of factors that can predict any long complications can affect the early treatment that can improve the outcomes. A study in Indonesia found that sex and necrotizing enterocolitis (NEC) staging might affect the survival of neonates with NEC, with female patients, who had a 3.1-fold higher risk of mortality than male patients. Moreover, patients with advanced stages of NEC will have thrombocytopenia within 24–72 h of disease onset. This study showed that NEC staging should be closely monitored and intervened as early as necessary Siahaan et al., Yuan et al.

There were also some case reports, animal studies, systematic review and meta-analyses that contributed to the growth of knowledge in the pediatric surgery field. A case report and literature review about primary omental lipoma in a pediatric case reported that although its etiology and pathology are unclear, the prognosis can be favorable with ultrasonography, CT, and MRI scanning for diagnosis and preoperative evaluation. The primary treatment is using laparoscopic surgery, and the prognosis is good Yuan et al. Another case report and literature review reported a case of idiopathic congenital abdominal aortic aneurysm. They conducted computed tomography angiography to reveal an isolated infrarenal abdominal aortic aneurysm and repaired it with artificial graft transplantation to prevent rupture. No genetic mutation was revealed in whole exome sequencing, and the outcome is favorable 40 months after surgery Zhou et al.

There was also a study that used an animal model in the form of gestated rabbits to study the use of oral spermine supplementation on villi height of immature intestines. Intestinal maturity in neonates due to premature birth was associated with a high risk of gut-derived infection and mortality. A morphological examination of hematoxylin-eosin-stained villi was performed. They found that oral spermine supplementation might improve intestinal villi height in immature intestines during gestation, achieving a mature newborn's height Tamba and Moenadjat.

Finally, some systematic reviews and meta-analyses were also published in this research topic. A team in China conducted a systematic review and meta-analysis of the remission effect of first- injection sclerotherapy for pediatric rectal prolapse, which is a common issue in clinical practice. They found that despite significant heterogeneity and low quality of evidence, the available data showed that the first injection of sclerotherapy might demonstrate therapeutic effects in achieving remission status in pediatric rectal prolapse patients Zhou et al. Another meta-analysis investigated the efficacy of polidocanol against venous malformations (VMs). They found polidocanol is a safe and effective treatment for VMs on different sides Liu et al.

The advancement of pediatric surgery is rapidly developing all around the world. Either in diagnosis and surgical treatment, the use of minimally invasive techniques and biomarkers to predict outcomes to future technologies such as robotic surgery. Keeping updated with the latest knowledge is essential for every clinician, researcher, and academic to finally be able to provide the best management for the well-being of patients.

